# Modeling the impact of the 7-valent pneumococcal conjugate vaccine in Chinese infants: an economic analysis of a compulsory vaccination

**DOI:** 10.1186/1472-6963-14-56

**Published:** 2014-02-07

**Authors:** Datian Che, Hua Zhou, Jinchun He, Bin Wu

**Affiliations:** 1 Department of Respiratory Disease, Shanghai Children’s Hospital, affiliated with the School of Medicine, Shanghai Jiaotong University, Shanghai, China; 2 Department of Emergency Care, Wuxi Children’s Hospital, affiliated with Nanjing Medical University, Shanghai, China; 3 Department of Otorhinolaryngology, Shanghai First People’s Hospital, affiliated with the School of Medicine, Shanghai Jiaotong University, Shanghai, China; 4 Medical Decision and Economic Group, Department of Pharmacy, Renji Hospital, affiliated with the School of Medicine, Shanghai Jiaotong University, Shanghai, China

**Keywords:** Cost-effectiveness analysis, PCV-7, Pneumococcal disease, Vaccine

## Abstract

**Background:**

The purpose of this study was to compare, from a Chinese societal perspective, the projected health benefits, costs, and cost-effectiveness of adding pneumococcal conjugate heptavalent vaccine (PCV-7) to the routine compulsory child immunization schedule.

**Methods:**

A decision-tree model, with data and assumptions adapted for relevance to China, was developed to project the health outcomes of PCV-7 vaccination (compared with no vaccination) over a 5-year period as well as a lifetime. The vaccinated birth cohort included 16,000,000 children in China. A 2 + 1 dose schedule at US$136.51 per vaccine dose was used in the base-case analysis. One-way sensitivity analysis was used to test the robustness of the model. The impact of a net indirect effect (herd immunity) was evaluated. Outcomes are presented in terms of the saved disease burden, costs, quality-adjusted life years (QALYs) and incremental cost-effectiveness ratio.

**Results:**

In a Chinese birth cohort, a PCV-7 vaccination program would reduce the number of pneumococcus-related infections by at least 32% and would prevent 2,682 deaths in the first 5 years of life, saving $1,190 million in total costs and gaining an additional 9,895 QALYs (discounted by 3%). The incremental cost per QALY was estimated to be $530,354. When herd immunity was taken into account, the cost per QALY was estimated to be $95,319. The robustness of the model was influenced mainly by the PCV-7 cost per dose, effectiveness herd immunity and incidence of pneumococcal diseases. With and without herd immunity, the break-even costs in China were $29.05 and $25.87, respectively.

**Conclusions:**

Compulsory routine infant vaccination with PCV-7 is projected to substantially reduce pneumococcal disease morbidity, mortality, and related costs in China. However, a universal vaccination program with PCV-7 is not cost-effective at the willingness-to-pay threshold that is currently recommended for China by the World Health Organization.

## Background

*Streptococcus pneumoniae* (*S. pneumoniae*) is the single most significant bacterial cause of invasive (meningitis and bacteremia) and noninvasive (pneumonia and otitis media) diseases in children <5 years of age worldwide [1]. In China, pneumococcal diseases pose a major burden in young children and the elderly, leading to nearly 30,000 deaths annually [2-4]. Pneumococcal diseases caused by *S. pneumoniae* are primarily treated with penicillin; however, the increasing prevalence of drug-resistant pneumococci is a concern worldwide and demands more sophisticated disease management. The treatment of infections caused by antibiotic-resistant *S. pneumoniae* would require more resources, including more expensive antibiotic agents and longer hospital stays, which would substantially increase healthcare expenditures [5-7].

New pneumococcal conjugate vaccines have been developed to protect infants and young children from pneumococcal diseases [8,9]. The heptavalent pneumococcal conjugate vaccine (PCV-7) Prevnar^®^ (Pfizer Vaccines) was the first such vaccine licensed by the U.S. Food and Drug Administration, and it has been available since 2000. PCV-7 is composed of seven saccharides from the capsular antigen of *S. pneumoniae*, each conjugated to a CRM_197_ protein, which is a nontoxic mutant of diphtheria toxin carrier. PCV-7 contains the serotypes 4, 6B, 9V, 14, 18C, 19F, and 23F, which cause the majority of cases of invasive pneumococcal disease (IPD) worldwide [10-12]. The clinical effectiveness and safety of PCV-7 in infants and children have been evaluated in several large-scale clinical trials. The results demonstrate that vaccination significantly decreased the incidence rates of IPD, pneumonia, and otitis media [13-15]. Furthermore, surveillance data indicated that PCV-7 could reduce the incidence rate of pneumococcal disease in both vaccinated infants and in unvaccinated infants by “herd immunity”. The direct effect of the vaccine could also extend the benefit to adults by decreasing the nasopharyngeal presence of *S. pneumoniae* in vaccinated children [16-18]. At present, new vaccines of higher valence, namely, a 10-valent vaccine (PCV-10) called Synflorix® (GlaxoSmithKline) and a 13-valent vaccine (PCV-13) (Pfizer Vaccines), have been approved to replace PCV-7 in the future.

Following recommendations from the Strategic Advisory Group of Experts on Immunization, the World Health Organization (WHO) suggests that the pneumococcal conjugate vaccine should be included in national immunization programs to reduce the heavy pneumococcal disease burden [19]. Over the last decade, many developed countries have added a pneumococcal vaccine to compulsory, routine immunization schedules [20]. In poor countries, multilateral organizations, including the Global Alliance for Vaccines and Immunization (GAVI), have taken an active role in improving access to vaccines [21]. At present, PCV-7 is the only pneumococcal conjugate vaccine licensed for use in infants and young children in China; however, the introduction of PCV-7 into the Chinese national immunization program still poses a challenge due to the high cost of the imported vaccine. The economic outcome of public health interventions is an important factor in policy decisions. Although economic outcomes in developed countries indicate that a universal PCV-7 vaccination program could reduce both medical and nonmedical costs, there is little economic evidence to support the universal use of this vaccine in China [22]. The aim of the present study was to evaluate the projected health benefits, costs, and cost-effectiveness of universal infant vaccination with PCV-7 in China.

## Methods

### Analytical and model overview

We used a decision tree-based mathematical model to estimate the costs and epidemiological impact of various vaccination scenarios [23]. The generic statistical model was programmed in R (version 2.14.1; R Development Core Team, Vienna, Austria) to project pneumococcal disease outcomes from birth to death for a hypothetical Chinese birth cohort of 16 million infants. A 5-year period was used because the effectiveness of this vaccine has yet to be determined. We assumed that each newborn entered the model healthy but at risk of incurring pneumococcal disease and developing adverse health outcomes. To estimate the long-term impact of pneumococcal disease, we extrapolated the lifetime outcomes of the individuals in the cohort (that is, until death at a maximum of 100 years of age). When indirect effects (herd immunity) are included in the model, adults in the family are considered to be at risk for developing IPD.

We estimated the impact of PCV-7 vaccination strategies compared to the current practice (no vaccination schedule) in China to address primary major public health issue faced by Chinese policy-makers. Because several studies have shown that the efficacy of two infant doses plus a booster (2p + 1) was similar to that of 3 infant doses and a booster (3p + 1), we assumed an implementation of a 2p + 1 schedule, with vaccines given at 4, 6 and 12 months of age [24-26].

**Figure 1:**
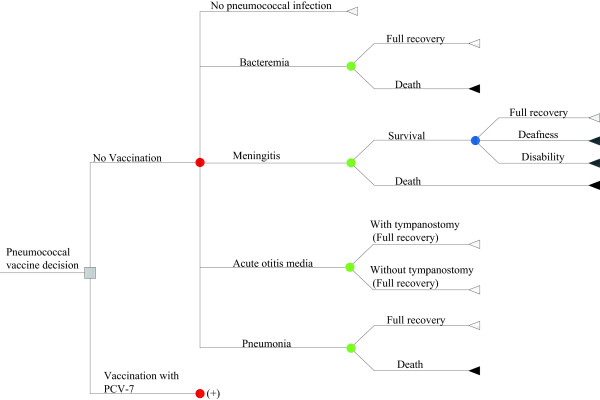
**The structure of the model, illustrating the two alternatives for PCV vaccination (including no vaccine) and possible subsequent events for each child.** The following health states are included: meningitis, bacteremia, pneumonia, AOM and no infection.

Initially, four pneumococcal diseases were included in the model: meningitis, bacteremia, community-acquired pneumonia (CAP) and acute otitis media (AOM). These four diseases can lead to complications, including tympanostomy, neurological hearing impairment, neurological disability, or death from pneumococcal disease. In the literature, the model is conceptualized as an extensively reported decision-tree framework that terminates in 11 mutually exclusive outcomes (Figure 1). The cycle lengths of the model in the time periods 0–4 years and 5–100 years are 1 month and 1 year, respectively. To simplify the model, adverse events were omitted from the analysis because pneumococcal conjugate vaccines are well tolerated and lead to only mild adverse events, such as injection-site reactions.

The analysis was modified to suit China, with costs in 2011 Chinese Yuan (CYN) converted into US dollars. Health outcomes were presented as the numbers of cases and deaths. Using the standardized methods recommended by the WHO, the estimated health outcomes were translated into gained quality-adjusted life years (QALYs). The incremental cost per QALY gained was used as the primary outcome. A yearly discount rate of 3% was used for both costs and health benefits. Univariate and probabilistic sensitivity analyses were performed to explore parameter uncertainty.

### Epidemiological and disease data

Model inputs for epidemiology data were obtained from Chinese studies to the greatest possible extent; however, the paucity of epidemiological pneumococcal disease studies in China necessitated the use of data from other countries, especially from East Asia (Table 1). The East Asian countries are in geographical proximity, and East Asian people are genetically and culturally related. These commonalities should theoretically result in similar serotypes and transmission patterns of *S. pneumoniae*. By searching the PubMed and Medline databases (up to January 26, 2012), we identified applicable articles on IPD in East Asia.

**Table 1:** Probabilities and estimates associated with pneumococcal disease and vaccine efficacy in Chinese children ≤ 5 years of age

**Variables**	Base-case estimate*	**Ranges for sensitivity analysis**	**Source**
Annual incidence rates of disease			
Pneumococcal meningitis (<2 years)	0.000051	Base ± 25%	[27]
IPD (3-5 years)	0.000142	0.000128–0.000156	[28]
All-cause pneumonia	0.12815	Base ± 25%	[2]
All-cause AOM (≤2 years)	0.412	0.012–0.64	[29]
All-cause AOM (3-5 years)	0.381	0.012–0.64	[29]
Proportion of meningitis in IPD	0.3333	Base ± 25%	[30]
*S. pneumoniae* isolation rate for pneumonia	0.08	0.02–0.268	[31]
*S. pneumoniae* isolation rate for AOM	0.392	0.292–0.49	[32]
Disease outcomes			
Case fatality rate of meningitis	0.083	Base ± 25%	[2]
Case fatality rate of bacteremia	0.046	Base ± 25%	[33]
Case fatality rate of pneumonia	0.00526	Base ± 25%	[2]
Probability of disability caused by meningitis	0.07	Base ± 25%	[34]
Probability of deafness caused by meningitis	0.13	Base ± 25%	[34]
Probability of tympanostomy caused by AOM	0.059	Base ± 25%	[13,35]
Serotype coverage rates of PCV-7			
IPD	0.45	Base ± 25%	[27]
Pneumonia	0.763	Base ± 25%	[36]
AOM	0.648	Base ± 25%	[37]
Efficacy of PCV-7			
IPD	0.974	Base ± 25%	[14]
Pneumonia	0.9	Base ± 25%	[14]
AOM	0.576	Base ± 25%	[15]
Relative efficacy of 2 doses	0.86	0.645–1	[38]
Relative efficacy of 3 doses	1	0.95–1	[24]
Birth cohort size in China	16,000,000	-	[39]

Age-specific incidence rates for pediatric cases of 4 pneumococcal diseases were derived from the published literature. The mean incidence rate of pneumococcal meningitis was estimated from a surveillance study of invasive bacterial diseases in Chinese children aged 1–23 months [27]. Additionally, a systematic review of IPD in East Asia was conducted to assess the probable incidence rate of pneumococcal meningitis in Chinese children >2 years of age. The surveillance data from Hong Kong, Taiwan, Singapore and Japan indicated that the mean incidence of IPD was 12.8–15.6 per 100,000 people per year [28]. We used the mean value of the above range as the assumed incidence rate of IPD in Chinese children aged 2–5 years. Based on an epidemiological study of IPD in China, the proportional incidence of meningitis in IPD is approximately 33.3%, and the proportional incidence of bacteremia is 66.7% [30]. Thus, the incidence rate of meningitis could be calculated by the following formula: 33.3% × incidence rate of IPD. The case-fatality rate of meningitis was derived from a systematic review published by Ying Chen *et al.*[2]. Two types of complications from pneumococcal meningitis (neurologic sequelae and deafness) were included in the model, and the risks of neurologic sequelae and deafness were 7.0 and 13.0%, respectively [34].

Due to the lack of incidence data on bacteremia in Chinese children aged 0-5 years, an indirect method was used to estimate the incidence rate. As the above Chinese study indicates that the incidence ratio of bacteremia to meningitis is nearly 2:1, the incidence rate of pneumococcal bacteremia for children aged 1–23 months was calculated as two times the incidence rate of pneumococcal meningitis. For children aged 2–5 years, the incidence rate of pneumococcal bacteremia was calculated as 66.7% of the incidence rate of IPD. The case-fatality rate of pneumococcal bacteremia was obtained from studies reported in Korea and Hong Kong [2,33,37].

No epidemiological study that determined the burden of pneumococcal pneumonia in China has been reported. A systematic literature review by Ying Chen *et al.* suggested that there were 12,815 cases of all-cause pneumonia per 100,000 people per year among Chinese children aged <59 months, including 384.5 cases of pneumococcal pneumonia with a 3% *S. pneumoniae* isolation rate for pneumonia [2]. However, due to the common practice of administering antibiotics prior to obtaining cultures, and as a result of poor culture methods, the proportion of positive blood cultures or CSF specimens is lower than expected. This scenario has implications for the current study because precise estimates of the disease burden would be highly sensitive to the true *S. pneumoniae* isolation rate. To avoid underestimating the isolation rate, we used the estimated isolation rate by O’Brien *et al.* The impact of the *S. pneumoniae* isolation rate on the outcome of the model was evaluated in a sensitivity analysis [31]. The mortality rate from all-cause pneumonia was derived from the systematic review published by Ying Chen *et al.*[2].

For AOM, the annual age-specific incidence data in children were derived from a study in a healthcare setting in Taiwan, China, in 2010 [29]. The reported annual incidence of acute otitis media was substantially different, ranging from 1.2% in an Asian population to 64% in a Finnish population. Because the incidence of acute otitis media is an important factor in the final economic analysis, we tested the impact of this input using a sensitivity analysis. The age-specific proportion of cases due to *S. pneumoniae* was obtained from a Chinese study by Wen RJ *et al.*, who investigated the distribution of pathogenic bacteria in 442 isolates from Chinese children of various ages with AOM [32]. The probability of tympanostomy was based on data from the United States and Finland [13,35]. As no reliable data were reported, the risk of sequelae caused by AOM was assumed to be zero.

### Vaccine efficacy and coverage

Pneumococcal conjugate vaccines were principally developed to cover the clinical pneumococcal serotypes encountered in North America and Western Europe, which are typically different from those found in China. The most common serotypes isolated from Chinese children with pneumococcal infections are 6A, 6B, 14,19F, 19A and 23F, while the serotypes covered by PCV-7 are 4, 6B, 9V, 14, 18C, 19F, and 23F. The estimated coverage rate of invasive isolates by PCV-7 was 45%, according to 2005–2006 surveillance data on children aged <5 years in eight different regions of China [27]. A recent prospective surveillance study of children aged <5 years in five different regions in China reported an estimated 76.3% coverage rate of PCV-7 for pneumonia [36]. Because there is no coverage of AOM in China, data from Korea were used. As the efficacy of PCV-7 in Chinese children is unknown and as there is no evidence that ethnicity affects the efficacy of PCV-7, the efficacy assumed in the current analysis was derived from the Northern California Kaiser Permanente (NCKP) trial and the Finnish Otitis Media Vaccine (Finnish OM) study [14,15]. The efficacies in the prevention of IPD, pneumonia and AOM caused by serotypes, which were covered by PCV-7, are 97.4, 90.0 and 57.6%, respectively [14,15]. As the vaccine coverage in the Chinese national vaccination program was close to 99%, we assumed the coverage of PCV-7 to be 99% [40]. The efficacy of partial vaccination was provided by the current model. We assumed that the efficacies of one and two doses alone are 0 and 86%, respectively, of the efficacy of the full three doses [38].

### Net indirect effect (herd immunity)

Studies have shown that the use of a pneumococcal conjugate vaccine in young children could markedly decrease the risk of pneumococcal disease in adults by diminishing nasopharyngeal vaccine serotypes (i.e., herd immunity) [41,42]. Recent studies have found that herd protection had a significant effect on the cost-effectiveness of a universal PCV7-vaccination program [43]. Herd immunity was included to calculate the effectiveness of vaccination in protecting unvaccinated children and adults for a period of 1 year, respectively. The incidence rates of IPD in individuals aged 0–4, 5–17, 18–49, 50–64 and 65+ years were 16.5, 1.4, 0.9, 4.2 and 12.1 per 100,000 persons, respectively, as reported in one recent study from a Chinese Taiwan district [44]. For children aged 4 years and younger, there was a 69% decrease in the presence of vaccine serotypes after the introduction of PCV-7 in the United Kingdom [45]. For four-dose vaccination schedules, reduction in IPD in the age groups 20–34, 35–64 and ≥65 years were 32% (95% CI: 23–41%), 8% (95% CI: 1–20%) and 18% (95% CI: 11–31%), respectively [42]. Because three-dose vaccination schedules are similar in direct efficacy to four-dose schedules, the net indirect effect was assumed to be the same for the two schedules. Herd protection did not affect AOM or CAP because there was no evidence supporting an indirect effect of vaccination on these diseases.

### Health outcomes

Vaccine efficacy was assessed by the following key parameters: specific disease cases, deaths averted and QALY gained. Health utility values were assigned to each specific health state in our model in a range from 1 (perfect health) to 0 (death). Utility values due to acute episodes of the disease and long-term sequelae are shown in Table 2 and were derived from previously published data [46-49].

**Table 2:** Cost* and utility of pneumococcal disease in Chinese children ≤5 years of age

**Variable**	**Base-case estimate**	**Ranges for sensitivity analysis**	**Source**
Cost			
Bacteremia per episode	2666.67	793.65–6349.21	Estimated
Meningitis per episode	3587.3	476.19–7936.51	Estimated
Pneumonia per episode	607.3	158.73–1587.3	Estimated
AOM per episode	111.11	31.75–317.46	Estimated
Tympanostomy caused by AOM	333.33	Base ± 25%	Estimated
Hearing aids per unit	634.92	300–1200	Estimated
Replacement interval (years)	6	3–10	Estimated
Special education for disability per year	2557.84	158.73–3174.6	Estimated
PCV-7 cost per dose	136.51	102.38–170.63	Estimated
Cost of vaccine administration	1.86	0–3.17	Estimated
Salary per day	22.6	6.3–27.64	Estimated
Work-lost days for disease			
Bacteremia	8	6–10	Estimated
Meningitis	9	6–12	Estimated
Pneumonia	7	5–9	Estimated
AOM	5	4–8	Estimated
Utility			
Bacteremia	0.9921	Base ± 25%	[46-49]
Meningitis	0.9768	Base ± 25%	[46-49]
Pneumonia	0.9921	Base ± 25%	[46-49]
AOM	0.995	Base ± 25%	[46-49]
Tympanostomy caused by AOM	0.82	Base ± 25%	[46-49]
Long-term disability	0.6	Base ± 25%	[46-49]
Long-term deafness	0.8	Base ± 25%	[46-49]
Death	0	-	[46-49]

*Costs are presented in US dollars (January 2012 exchange rate, US$ = CYN 6.30).

### Resource Use and costs

The cost associated with pneumococcal disease was determined from a Chinese perspective. The medical and nonmedical cost parameters used in this analysis are presented in Table 1. For the four initial diseases, the assumed medical costs were directly associated with treatment for pneumococcal disease in Shanghai Children’s Hospital, Wuxi Children’s Hospital and Shanghai First People’s Hospital in 2011. These costs were adjusted based on the opinions of a Chinese pediatric expert panel. Cases with other severe comorbidities, such as malignant cancer, immune disorders and congenital cardiac or respiratory diseases, were excluded from the cost estimates. The medical costs covered the medical resources consumed in the treatment of pneumococcal disease, such as medications, medical care, hospitalization and diagnostic tests. The cost of productivity lost by a parent taking care of a child with pneumococcal disease was estimated by an expert panel. The median daily salary in China ($22.6 according to the Chinese National Bureau of Statistics) was used to estimate the total opportunity cost of pneumococcal disease by multiplying work-loss by time [50].

The costs of the long-lasting sequelae of meningitis, including deafness and neurologic impairment, were included in this analysis. As described by Butler JR *et al.*, the additional resource consumption for deafness is one hearing aid per a designated number of years for the remainder of a patient’s life [51]. The median time and cost to replace a disabled hearing aid is 6 years and $635, respectively, according to the expert panel. Ranges of 3–10 years and $300–1200 were used in the sensitivity analysis. Individuals with neurological deficits commonly require special education and lifetime residential care, which were included as an additional cost. We assumed that, in accordance with Chinese education law, children with disabilities would receive 9 years of special education. The annual cost of special education was derived from the Statistical Yearbook of Chinese Education [52]. To our knowledge, care in China is always the responsibility of a family member or members. Thus, we estimated the cost of care based on the lost productivity of a family member. The average wage in China was used to calculate the potential annual economic burden of neurological deficit [50].

The cost of adding PCV-7 to the vaccination program included both PCV-7 acquisition and non-vaccine costs. The per-dose retail price of PCV-7 in China is $127; however, a discount may be possible when drugs and vaccines are covered by the Chinese healthcare system. To account for changing price politics in China, a sensitivity analysis on this input variable was conducted. Non-vaccine costs included administration, transport, injection supplies, training, and other expenses. In the current analysis, we assumed that the non-vaccine costs associated with PCV-7 per dose were similar to those associated with the H1N1 vaccine ($1.86) [53].

### Sensitivity analysis

Univariate and probabilistic sensitivity analyses were performed to examine the uncertainty within the model [54]. In the univariate sensitivity analyses, the parameters were varied to evaluate the sensitivity of the findings in the presence of plausible variations in specific data inputs. The results of the univariate sensitivity analyses are presented in a Tornado diagram. The ranges of parameters used in the univariate sensitivity analyses were obtained from published literature; when reported data were not available, a range of ±25% of the base-case value was used (Tables 1 and 2). The range in vaccine price per dose was assumed to be $102.38 to $170.63. For the probabilistic sensitivity analyses, parameters were sampled using the Monte Carlo method to simulate 1,000 replication outcomes. The beta distribution was used for incidence rates, risks, probabilities, proportions and utilities, and the gamma distribution was used for costs. Cost-effectiveness acceptability curves (CEACs) of vaccination versus no vaccination were developed to present the probabilities of cost-effectiveness at different costs of PCV-7.

## Results

### Base-case analyses

The projected health outcomes and costs for the “no vaccination” and PCV-7 strategies in China are presented in Table 3, with listed differences between the two strategies. Over a 5-year period, the model predicted that universal PCV-7 vaccination in the Chinese birth cohort would prevent 4,222 cases of IPD, 4,061,524 cases of AOM and 472,527 cases of pneumonia, preventing an additional 2,682 deaths from pneumococcal disease. PCV-7 vaccination was estimated to prevent 235,819 cases of tympanostomy from AOM, as well as 97 cases of permanent disability and 180 cases of deafness from meningitis. Overall, it was projected that PCV-7 vaccination in a cohort of nearly 16,000,000 Chinese infants would lead to the addition of 9,895 QALYs (discounted by 3%) over the lifetime of the infants. Accounting for herd immunity, 16,018 cases of IPD and 1,515 deaths in the adult population would be prevented by PCV-7 vaccination, which translates into an additional 43,543 QALYs (discounted by 3%) gained over the lifetime of the vaccinated infants.

**Table 3:** Projected health outcomes and costs of pneumococcal conjugate vaccination in a cohort of Chinese infants on lifetime disease burden compared with no vaccination

**Disease type/health outcome**	**No vaccination**	**PCV-7 Vaccination**	Differences*
Cases for birth cohort			
Pneumococcal bacteremia	7,402	4,587	-2,815
Pneumococcal meningitis	3,701	2,293	-1,407
Pneumococcal AOM	12,616,500	8,554,976	-4,061,524
Pneumococcal pneumonia	780,407	307,880	-472,527
Disability caused by pneumococcal meningitis	255	158	-97
Deafness caused by pneumococcal meningitis	474	294	-180
Tube insertion caused by pneumococcal AOM	732,905	497,086	-235,819
Death caused by pneumococcal disease	4,674	1,993	-2,682
Death caused by pneumococcal bacteremia	335	207	-128
Death caused by pneumococcal meningitis	302	188	-114
Death caused by pneumococcal pneumonia	4,037	1,597	-2,439
Efficacy of herd immunity			
Pneumococcal bacteremia	28,310	23,562	-4,748
Pneumococcal meningitis	19,088	7,817	-11,270
Disability caused by pneumococcal meningitis	3,627	1,485	-2,141
Deafness caused by pneumococcal meningitis	4,963	2,033	-2,930
Death caused by pneumococcal invasive disease	5,449	3,934	-1,515
Costs for birth cohort ($, discounted)			
vaccine cost (including administration)	0	6,438,882,934	6,438,882,934
Meningitis medical cost	13,275,258	8,226,631	-5,048,627
Bacteremia medical cost	19,739,872	12,232,718	-7,507,154
Pneumonia medical cost	473,942,531	186,976,189	-286,966,342
AOM medical cost	1,401,833,288	950,552,900	-451,280,388
Long-term cost of sequelae	6,428,514	3,974,969	-2,453,545
Nonmedical cost	1,581,876,301	1,144,173,592	-437,702,709
Subtotal	3,497,095,763	8,745,019,933	5,247,924,169
Costs in herd immunity	323,840,533	169,576,767	-154,263,765
Total	3,820,936,296	8,914,596,700	5,093,660,404
QALYs loss (without herd immunity)	2,310,183	2,300,288	43,543^#^
QALYs loss (with herd immunity)	112,072,439	112,019,001	53,438^#^
ICER without heard immunity ($/QALY gained)		530,354	
ICER with heard immunity ($/QALY avoided)		95,319	

*PCV-7 vaccination strategy compared with no vaccination.

#The incremental QALYs gained by PCV-7 vaccination strategy.

The medical and nonmedical cost of pneumococcal disease was estimated to be $3.5 billion (discounted by 3%) in an unvaccinated birth cohort; this value was driven by the nonmedical cost and the costs associated with AOM. The inclusion of the costs associated with IPD in unvaccinated populations increased the total to almost $3.8 billion (Table 3). A universal PCV-7 vaccination program would save $1,345 million in costs associated with pneumococcal disease, including savings in the Chinese birth cohort ($1,190 million) and herd immunity for unvaccinated populations ($154 million). The cost of implementing a 3-dose PCV-7 vaccination schedule for a birth cohort of nearly 16,000,000 Chinese infants was estimated to be $6.44 billion.

From a societal perspective, the incremental cost-effectiveness ratio of universal PCV-7 vaccination over no vaccination was $530,354 per QALY gained. When the indirect effect of herd immunity from vaccination was taken into account, the cost per QALY gained was $95,319 (Table 3).

### Sensitivity analyses

**Figure 2:**
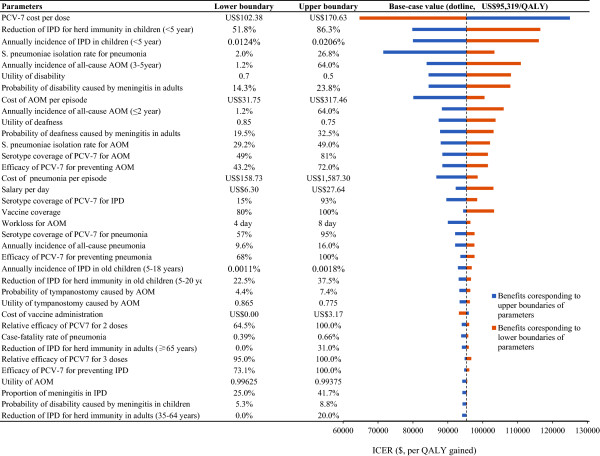
**Tornado diagram presenting a one-way sensitivity analysis for PCV-7 vaccination compared with no vaccination.** The length of the bars represents the differences in ICERs with low (left) and high (right) parameter values.

The results of the one-way sensitivity analyses are shown in a tornado diagram (Figure 2), which presents the 37 most sensitive input variables around the base-case value in terms of the discounted ICER (≥1% of base-case ICER and listed in descending order). The projected ICER of PCV-7 vaccination versus no vaccination was most sensitive to the cost of PCV-7 per dose. The cost varied from $102.38 to $170.63, and the corresponding ICERs were $64,711 and $124,957, respectively. The reduction of IPD for herd immunity in adults (<5 year) and annual incidence of IPD in children (<5 year) were the second- and third-most influential factors, respectively. As expected, a higher *S. pneumoniae* isolation rate for pneumonia predicted more favorable ICERs.

**Figure 3:**
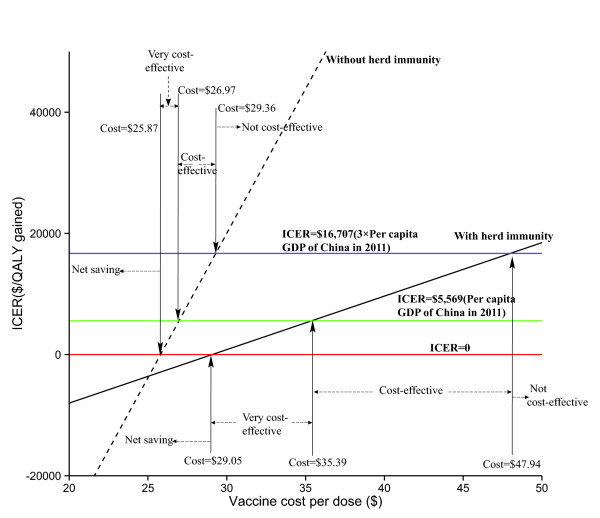
Projected cost per QALY gained by the PCV-7 vaccination program for healthy Chinese infants as a function of vaccine cost per dose with or without herd immunity.

In China, the break-even costs of the PCV-7 vaccine with and without herd immunity were $25.87 and $29.05, respectively (Figure 3). When herd immunity was taken into account, the per-vaccine cost ranges for very cost-effective, cost-effective and not cost-effective vaccination were $29.05 to $35.39, $35.39 to $47.94 and ≥ $47.94, respectively. When herd immunity was not taken into account, the relevant per-vaccine cost ranges were $25.87 to $26.97, $26.97 to $29.36 and ≥ $29.36, respectively.

The results were moderately sensitive to the cost of AOM per episode, the annual incidence of all-cause AOM in children, the serotype coverage of PCV-7 for IPD and the *S. pneumoniae* isolation rate for AOM. Other moderately sensitive factors include the *S. pneumoniae* isolation rate for AOM, serotype coverage of PCV-7 for AOM, cost of AOM per episode, efficacy of PCV-7 at preventing AOM and pneumonia and reduction of IPD in adults (≥65 year) with herd immunity. Other input model parameters, such as the cost of meningitis and bacteremia per episode, had little impact on projected ICERs (<1% of base-case ICERs).

**Figure 4:**
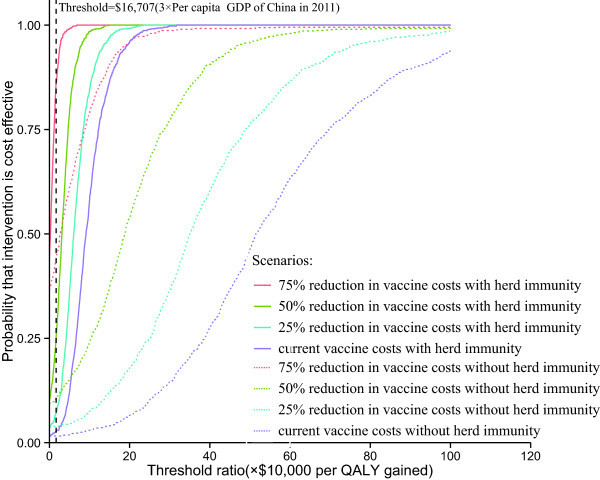
Cost-effectiveness acceptability curves of base-case vaccination schedules and of alternative scenarios regarding PCV-7 price.

Finally, given the willingness to pay $5,569 (per capita GDP of China in 2011) or $16,707 (three times the per-capita GDP of China in 2011) per QALY gained, cost-effectiveness acceptability curves showed that the probability that a vaccination plan will be cost-effective is less than 0.5, only when the cost was decreased 75% and herd immunity was taken into account (Figure 4).

## Discussion

Although the incidence rate in China is lower than in other developing regions, such as Africa and Southeast Asia, China accounts for 12% of pneumococcal cases worldwide because it has the largest population [31]. The introduction of PCV vaccines has increased interest in preventing pneumococcal disease. Our results show that the routine compulsory vaccination with PCV-7 of Chinese infants has the potential to significantly decrease the disease burden and mortality associated with *S. pneumoniae* by 33.8 and 57.4%, respectively. The surveillance data from the regions where PCV-7 has already been incorporated into the national immunization program demonstrate the effectiveness of this vaccine [16,55]. In the United States, a 75% decrease in the all-cause IPD incidence rate was observed within 3 years of vaccine introduction, and nearly all IPD caused by vaccine serotypes was prevented in children ≤5 years of age [55]. The estimated cost associated with pneumococcal disease was reduced by 34.1% in the birth cohort receiving PCV-7 vaccination. However, our economic analysis indicated that the PCV-7 vaccination program in China is not cost-effective because the incremental cost per QALY gained is far greater than three times the per-capita GDP of China, which is the standard cutoff cost recommended by the WHO Choosing Interventions that are Cost Effective (WHO-CHOICE) project. This result persisted even when a 75% discount rate was applied to the per-dose price of PCV-7 [56,57]. To the best of our knowledge, this is the first cost-effectiveness evaluation of the economic outcome of PCV-7 vaccination implementation in China. The results may be helpful for Chinese health-policy makers in deciding whether to add PCV-7 to the compulsory routine vaccination program in China.

Our results indicated that the cost of the vaccine itself drives the cost of PCV-7 vaccination, accounting for 72.2 or 73.6% of the total cost with or without herd immunity, respectively. The one-way sensitivity analysis showed that varying the cost of PCV-7 from 75 to 125% of the base-case value yielded the most influential parameters. The literature has shown that the cost of PCV-7 vaccine acquisition in the rest of the world is substantially different from the cost in China. For example, the estimated current cost of PCV-7 per dose in the European national immunization program was nearly $80, which is 41% lower than the cost in China; [58] however, only a 75% reduction in vaccine cost would yield a nearly 75% probability of cost-effectiveness when herd immunity was considered (Figure 4). To satisfy the standard of cost-effectiveness, Figure 3 suggests that the cost of PCV-7 should be reduced to at least 35.1 or 21.5% of the base-case value with or without herd immunity, respectively. This suggestion is in accordance with the study by Mari Nakamura *et al.*, which indicated that vaccination with PCV-7 would be cost-effective at a per-dose cost of $10 for lower-middle-income (2008 GNI per capita: $976–3,855) and $20 for upper-middle-income (2008 GNI per capita: $3,856–11,905) countries [59]. The costs of introducing a compulsory routine pneumococcal vaccination program in China should be weighed against the losses based on more information regarding budget impact, affordability and sustainability.

Our findings project a 38% reduction in IPD following PCV-7 vaccination in China, which is greater than the reduction in pneumococcal AOM (32%); however, our results indicate that the major contributor to the cost savings from the introduction of PCV-7 vaccination emerges from a reduction in costs related to pneumococcal AOM. Otitis media has relatively less serious outcomes and a higher incidence rate compared with IPD according to this analysis, in agreement with the findings of previous studies. Pneumococcal non-typeable *Haemophilus influenza* protein D conjugate vaccine (PHiD-CV) is a newly licensed 10-valent pneumococcal conjugate vaccine. It may protect against diseases caused by non-typeable *H. influenzae* (NTHi) [60]. Vaccination with PHiD-CV is projected to lead to a 33.6% decrease in the overall incidence of otitis media and a 35.6% reduction in the incidence of otitis media caused by *H. influenzae*[60]. It is expected that a more favorable ICER would be achieved if the cost of PHiD-CV were comparable to that of PCV-7. The base-case analysis also showed that the cost associated with pneumococcal pneumonia was the second major contributor to the total economic burden of pneumococcal disease. According to a recent study by Kai-Hu Yao *et al.*, serotype 19A, which is not covered by PCV-7, is the second most common serotype in Chinese children [36]. The serotype coverage rate of PCV-13, which does include 19A, for pneumococcal pneumonia is 16% higher than that of PCV-7. New pneumococcal vaccines with higher serotype coverage rates should be given serious consideration when deciding whether to support pneumococcal vaccination in China. The current analysis should be updated when PCV-10, PCV-13 and PHiD-CV are supplied in China.

Previous studies have shown that the addition of indirect effects offer considerable support for the universal vaccination of young children [43]. Although efforts to introduce PCVs in developing countries are increasing, few published reports have used local data to account for the role of herd immunity from universal childhood PCV-7 vaccination in reducing adult cases of IPD. The evidence from early epidemiological studies showed that herd immunity decreases the overall burden of pneumococcal disease [41,55]. In comparison to a model that does not account for herd immunity from universal infant PCV-7 vaccination in China in terms of reducing adult cases of IPD, our base-case analysis indicated that the addition of this indirect effect yielded a substantially lower ICER (Table 3). The results of the current analysis consistently demonstrated a more favorable economic outcome when herd immunity was included in the vaccination decision model; however, it should be noted that the health-economic benefits of PCV-7 would be reduced by serotype replacement with uncovered virulent strains. Recent epidemiological data from the United States, Europe and Singapore suggest ongoing and significant serotype changes in children, with 19A in particular increasing in incidence [61-63]. The adoption of PCVs with broader serotype coverage is strongly recommended. Meanwhile, continued surveillance of IPD is necessary to provide epidemiological data on potentially emerging serotypes.

The present analysis took a conservative approach to estimating the health benefits of PCV-7 vaccination for three reasons. First, the increasing frequency and rapid spread of antibiotic-resistant *S. pneumoniae* is a global problem, and serious antibiotic resistance has been observed in Chinese clinical practice. According to an epidemiological study by Lin Zhou *et al.*, the rates of resistance to erythromycin and azithromycin in *S. pneumoniae* in Beijing were 96.4 and 97.1%, respectively. Furthermore, 64.3% of all pneumococcal isolates were multidrug-resistant *S. pneumoniae* (MDRSP) [64]. To manage pneumococcal disease caused by antibiotic-resistant *S. pneumoniae*, superior antibiotics and longer hospital stays are necessary, leading to increased consumption of health-associated financial resources. In the present analysis, resource savings from the prevention of antibiotic-resistant infection were not accounted for among the potential savings from PCVs because local data on the subject are inadequate and difficult to collect. The exclusion of such infections underestimates the cost-effectiveness of universal PCV-7 vaccination in China. Second, the most influential factor captured by our model was the *S. pneumoniae* isolation rate for pneumonia. Our findings indicated that a higher isolation rate would yield more favorable health benefits from vaccination. The isolation rate used in the base-case analysis were 8%, which is greater than the estimates provided by Ying Chen *et al.*; [2] however, as a result of widespread antibiotic abuse in China, pathogen cultures from blood and cerebrospinal fluid (CSF) are rarely positive, and nasopharyngeal isolates are the main method of surveillance for pneumococcal epidemiology in Chinese children. According to a study by Rudan *et al.*, the estimated proportion of pneumococcal pneumonia in all-cause radiological and fatal pneumonia was 30–50% in Chinese children aged ≤5 years [65]. One recent study conducted in Beijing found that nearly 55% of children with severe community-acquired pneumonia had *S. pneumoniae* in their lung tissues, as identified by PCR and/or Southern blotting [66]. Because of the limitations of these data, our model did not adjust for the isolation rate for *S. pneumoniae*. These conservative estimates may have led us to underestimate the impact of a PCV program. Finally, several studies have indicated that the benefits of PCV7 vaccination extend beyond the covered serotypes. A separate study found a 20% reduction in non-pneumonia acute respiratory infections in children aged ≤2 years after the adoption of PCV-7 [67]. Data from South Africa and the United States indicated that PCV may reduce the incidence of viral-associated pneumonia [68]; however, our model does not consider these broader benefits of the vaccine and thus underestimates the impact of the vaccine.

This study has several weaknesses and limitations. First, in the absence of an epidemiological survey, no China-specific data were available for some of the model inputs, and there was substantial variation in data quality. Thus, such results must be carefully interpreted because multiple sensitive variables limit the extrapolation of results (e.g., the incidence of all-cause AOM, *S. pneumoniae* isolation rate, and efficacy of herd immunity). Fortunately, our sensitivity analyses indicated that these variables do not significantly affect the ICERs in terms of the WHO-CHOICE standards. Second, to simplify the model structure and to avoid including too many uncertainties, the current model does not include all diseases associated with *S. pneumonia* and adverse reactions related to the vaccination as other economic analyses do, such as sinusitis, septic arthritis and injection-side reactions [21,22,69-71]. However, because the excluded diseases or events (e.g., seizure disorders and vision loss) are rare, their impact was too minor to be captured by the model. Third, almost all China-specific data in this analysis, such as the serotype coverage of PCV-7 for pneumonia, were obtained from hospital-based studies. These studies were mostly conducted in teaching hospitals in large cities such as Beijing, Shanghai and Guangzhou, where sanitary and health conditions are likely better than those in small cities and rural regions. Thus, such data may not be representative of the entire Chinese population, and there are uncertainties regarding the robustness of the analysis. If better supported and more representative epidemiological data become available in the future, the model outcome will be more accurate, and the relative effectiveness of the vaccines in preventing IPD will be greater. Fourth, potential uncertainties and biases result from the several assumptions and expert opinions used in this model, such as the efficacy of PCV-7 vaccination on a three-dose schedule and the productivity loss of parents who must care for their children. We adjusted these assumptions in the sensitivity analysis to determine the robustness of the model. Fifth, as other analyses have showed that herd immunity had a significant effect on cost-effectiveness, [43] it should be carefully explained that the current analysis used the indirect efficacy data from other countries, where the patterns of mixing and serotype may differ greatly from that of China. Finally, changes in health-resource consumption and quality of life due to vaccine-related adverse events were not included in the current analysis, which may lead to an overestimation of the cost of disease. The ICER for vaccination would have been more unfavorable if such expenditures had been included; however, the available evidence from large-scale studies indicates that PCV-7 was well tolerated and that the impact on cost and quality of life would be minimal. Despite these shortcomings, our analysis provides insight into the potential of PCVs to reduce the burden of pneumococcal disease.

## Conclusion

The addition of PCV-7 to the Chinese vaccination program could prevent a considerable number of pneumococcal diseases. The economic results of this analysis indicate that a vaccination strategy based on QALYs gained is not cost-effective in comparison with a no-vaccination strategy, even when accounting for herd immunity. The sensitivity analyses indicate that the model outcome is sensitive to some parameters, such as the price of PCV-7 and the incidence of pneumococcal diseases in China. Thus, to better inform healthcare decision-making, further work is needed to improve surveillance data in China. Furthermore, due to the high price of the PCV-7 vaccine and its partial efficacy when applied to the population of Chinese infants, other vaccination policies should be explored for improvement of health resource allocation. Children with high risk of developing pneumococcal diseases should be emphasized in the economic analysis if the non-compulsory vaccination is considered.

## Competing interests

Declaration of personal interests: All authors have nothing to declare.

## Authors’ contributions

BW contributed to the conception and design of the model and interpreted the results. DC developed the economic model, performed the analyses and drafted the manuscript. HZ and JH collected and reviewed data. All authors read and approved the final manuscript.

## Pre-publication history

The pre-publication history for this paper can be accessed here:

http://www.biomedcentral.com/1472-6963/14/56/prepub
